# Fumaric Acid Production in *Saccharomyces cerevisiae* by *In Silico* Aided Metabolic Engineering

**DOI:** 10.1371/journal.pone.0052086

**Published:** 2012-12-26

**Authors:** Guoqiang Xu, Wei Zou, Xiulai Chen, Nan Xu, Liming Liu, Jian Chen

**Affiliations:** 1 State Key Laboratory of Food Science and Technology, Jiangnan University, Wuxi, China; 2 The Key Laboratory of Industrial Biotechnology, Ministry of Education, School of Biotechnology, Jiangnan University, Wuxi, China; 3 The Key Laboratory of Carbohydrate Chemistry and Biotechnology, Ministry of Education, School of Biotechnology, Jiangnan University, Wuxi, China; Instituto Nacional de Cardiologia, Mexico

## Abstract

Fumaric acid (FA) is a promising biomass-derived building-block chemical. Bio-based FA production from renewable feedstock is a promising and sustainable alternative to petroleum-based chemical synthesis. Here we report on FA production by direct fermentation using metabolically engineered *Saccharomyces cerevisiae* with the aid of *in silico* analysis of a genome-scale metabolic model. First, *FUM1* was selected as the target gene on the basis of extensive literature mining. Flux balance analysis (FBA) revealed that *FUM1* deletion can lead to FA production and slightly lower growth of *S. cerevisiae*. The engineered *S. cerevisiae* strain obtained by deleting *FUM1* can produce FA up to a concentration of 610±31 mg L^–1^ without any apparent change in growth in fed-batch culture. FT-IR and ^1^H and ^13^C NMR spectra confirmed that FA was synthesized by the engineered *S. cerevisiae* strain. FBA identified pyruvate carboxylase as one of the factors limiting higher FA production. When the *RoPYC* gene was introduced, *S. cerevisiae* produced 1134±48 mg L^–1^ FA. Furthermore, the final engineered *S. cerevisiae* strain was able to produce 1675±52 mg L^–1^ FA in batch culture when the *SFC1* gene encoding a succinate–fumarate transporter was introduced. These results demonstrate that the model shows great predictive capability for metabolic engineering. Moreover, FA production in *S. cerevisiae* can be efficiently developed with the aid of *in silico* metabolic engineering.

## Introduction

Fumaric acid (FA) is widely used in food, pharmaceutical and chemical industries, and is attracting increasing attention because it can be converted into therapeutic drugs and is a starting material for polymerization and esterification. FA is mainly produced petrochemically from maleic anhydride at present. Increasing petroleum prices, concerns about climate change and advances in the field of metabolic engineering have fueled renewed interest in the production of organic acids by microbial fermentation [1]. Although high FA yields have been obtained from fungi such as Rhizopus oryzae [2] and Rhizopus arrhizus [3], the process might be limited at the industrial scale because these fungi are difficult to grow and their morphology can strongly affect production characteristics. The yeast Saccharomyces cerevisiae was regarded as a suitable microorganism for biotechnological production of carboxylic acids [4], and significant progress has been made in exploring metabolic engineering for the production of carboxylic acids such as lactic [5], malic [6], [7], and succinic acids [8], [9] by S. cerevisiae.

At least two metabolic strategies can be used for FA production by *S. cerevisiae*. In the first, FA can be produced via a reductive tricarboxylic acid (TCA) cycle, which provides a maximum theoretical yield of 2 moles of FA per mole of glucose. Moreover, this process involves biological CO_2_ fixation instead of release, which is of great interest because of increasing concerns about climate change. In our previous study, an exogenous fumarate biosynthetic pathway involving reductive reactions of the TCA cycle was successfully introduced in *S. cerevisiae* via a series of simple genetic modifications and pyruvate carboxylase was identified as one of the factors limiting fumarate production [10]. However, the energy balance for FA synthesis via a reductive TCA cycle is barely even and does not provide any ATP for maintenance and active transport processes, and the redox balance is uneven. In the second strategy, FA can be produced via an oxidative TCA cycle and the engineered strain is stable in the fermentation process. It was reported that cells of a fumarase-deficient mutant accumulated extracellular FA when fermenting glucose [11]. Similarly, a concentration of 3.62 g L^–1^ at a yield of 0.11 moles of succinic acid per mole of glucose was achieved for oxidative production of succinic acid in yeast by deletion of the *SDH1*, *SDH2*, *IDH1* and *IDP1* genes [8].

Recent advances in genomics and other -omics technologies combined with computational analysis have opened new avenues for strain improvement [12]–[15]. Metabolic engineering combined with systems biology has been successfully applied to the development of strains capable of enhanced production of chemicals and materials by redistributing and optimizing metabolic fluxes [16]. Identification of genes for manipulation is an essential step in metabolic engineering for strain improvement for enhanced production of target bioproducts.

**Figure 1:**
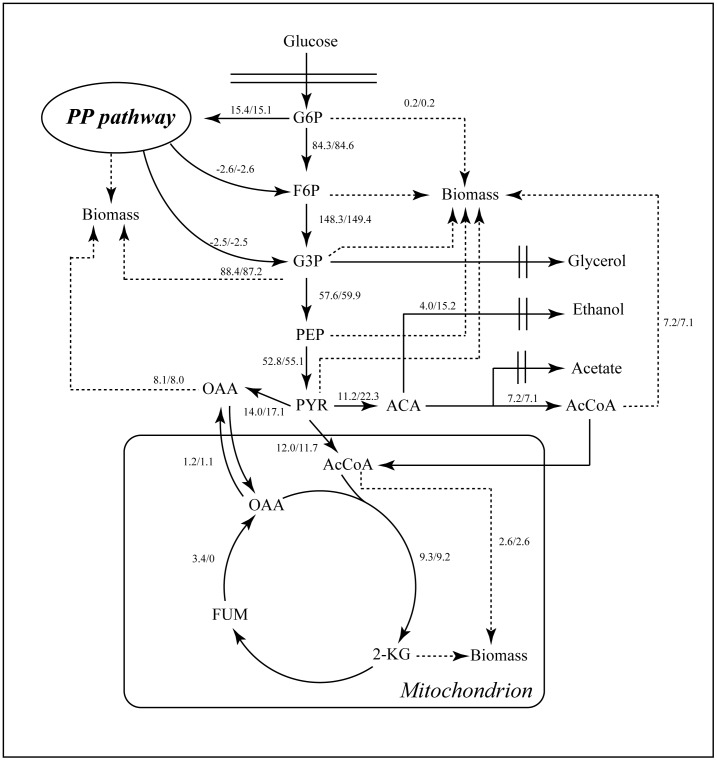
The major metabolic pathway leading to the formation of fumaric acid and *in silico* carbon flux distribution in the central metabolism of *S. cerevisiae* during fumaric acid production on glucose. a/b, “a” represent the flux of parent strain, “b” represent the flux of the mutant strain FMME-002Δ*FUM1*. Fluxes are shown relative to a glucose uptake rate of 100.

In the present study, the target gene for FA production in S. cerevisiae was identified via literature mining. Then iND750, a validated genome-scale metabolic model (GSMM) of S. cerevisiae [17], was used for in silico simulation of the metabolic response to deletion of the target gene by flux balance analysis (FBA) [18] and robustness analysis (Figure 1) [19]. Rational metabolic engineering [20] was then applied to develop a S. cerevisiae strain capable of efficient FA production. In addition, to further improve FA production, the model combined with literature surveys was used as a tool to indentify the controlling steps, and experimental validation was performed.

## Results

### Target Selection and *in silico* Simulation

To search for target genes for FA production in S. cerevisiae, extensive mining of the literature on FA and S. cerevisiae was carried out. The results of this literature survey revealed that fumarase defects or FUM1 deletion can lead to FA production. Thus, FUM1 was selected as the target gene to be manipulated.

**Figure 2:**
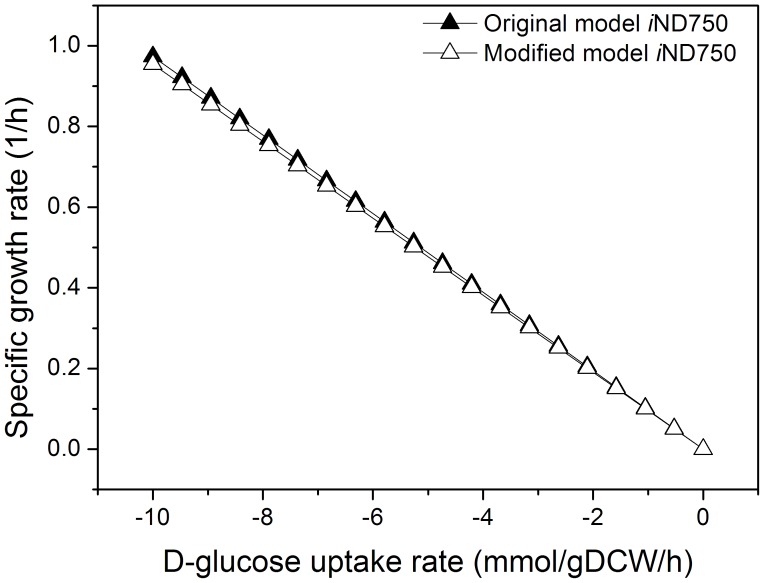
Robustness analysis for the D-glucose uptake rate and growth rate. Closed triangle represents the original model *i*ND750, open triangle represents the modified model *i*ND750.

FBA analysis revealed that FUM1 deletion can lead to FA production at a rate of 0.357 mmol g^–1^ DCW h^–1^ for the modified model, whereas FA was not produced in the original iND750 model. Robustness analysis of the rates of D-glucose uptake and growth for the original and modified models showed that FUM1 deletion leads to slightly lower growth of S. cerevisae (Figure 2); the growth rate predicted for the modified model (0.954 h^–1^) was only ∼1.95% lower than for iND750 (0.973 h^–1^). Thus, the in silico simulation indicated that FUM1 deletion should not only result in FA accumulation, but should also have no obvious influence on S. cerevisiae growth.

### Strain Construction, Aerobic Batch Fermentation and FA Characterization

The *fum1*-deleted mutant was obtained by double-crossover replacement. To confirm that the disruption cassette was correctly integrated in the genome and replaced the target *FUM1* gene, PCR analysis was performed using two primer sets (A and BM, and CM and D; Figure S1). To disrupt or overexpress other genes in a yeast strain for subsequent study, the gene disruption cassette can be removed from the genome so that the marker can be used a second time. Loss of the HIS marker was verified by appropriate PCR analysis using the primer sets A and BM, and CM and D (Figure S1).

**Figure 3:**
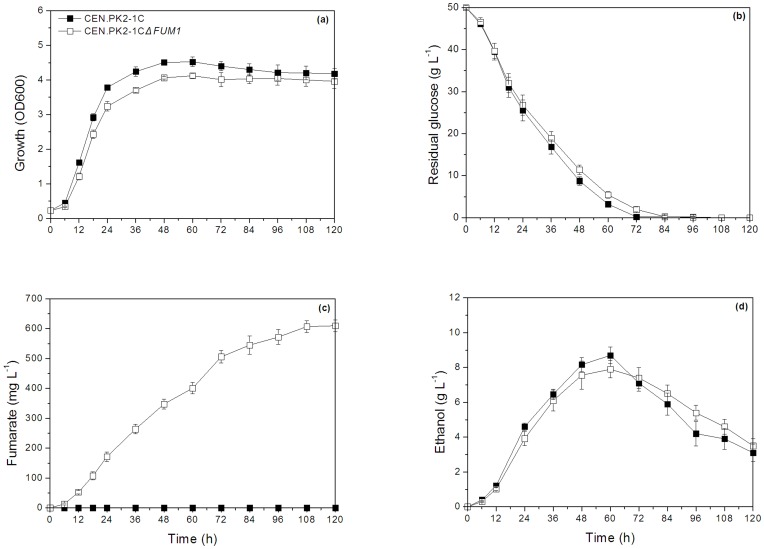
Fermentation profile for cell growth and product accumulation during aerobic batch culture. Closed square represents the parent strain, open square represents the mutant strain FMME-002Δ*FUM1*. (a): growth, (b): residual glucose, (c): fumaric acid, and (d): ethanol. Values are presented as means of three independent experiments. Bars represent the standard deviation.

To investigate the effects of *FUM1* deletion on FA production, time profiles for growth characteristics, glucose consumption, FA production and ethanol formation were compared for the parent strain and the mutant. The mutant showed a slightly lower growth rate and ethanol formation compared to the parent strain. Moreover, the mutant accumulated FA to a concentration of up to 610±31 mg L^–1^ (yield of 0.018 moles of FA per mole of glucose) after 120 h of cultivation, while no FA was detected in the broth of parent strain during fermentation (Figure 3). The results are similar to those predicted by the *S. cerevisiae i*ND750 GSMM.

The chemical structure of fumarate samples was confirmed by FT-IR and ^1^H and ^13^C NMR spectroscopy. IR spectra of samples and a fumarate standard were the same. Stretching vibrations of the carbonyl group were observed as strong bands at 1600 and 1850 cm^–1^, while stretching vibrations of the hydroxyl group were observed as broad bands at 3200 and 2500 cm^–1^(Figure S2). ^1^H NMR spectra of a sample and the reference standard are shown in Figure S3. The peak at 6.84 ppm was assigned to the CH proton, while the OH proton was substituted by deuterium from D_2_O. ^13^C NMR spectra of a fumarate sample and the reference standard are also shown in Figure S3. Both spectra show two signals, including two carboxylic carbons at 136.87 ppm, and two double-bonded carbons at 171.66 ppm. The results confirm that FA was synthesized by the engineered *S. cerevisiae* strain.

### Strategies for Improving FA Production

To further improve FA production, key factors (such as enzymes) preventing channeling of the carbon flux to FA must be identified. First, the central metabolic flux of CEN.PK2-1CΔFUM1 was analyzed with cell growth as the objective when FA production successively increased from the control value (0.4 mmol g DCW h^–1^) to the theoretical maximum value using iND750 and an FBA algorithm. According to the results, three types of intracellular flux profiles occur: increased, decreased and irregular (Table S1). As shown in Table 1, among the increased intracellular flux profiles, the carbon flux of pyruvate carboxylase increased more apparently with FA production, indicating that pyruvate carboxylase could be one of factors limiting higher FA production.

**Table 1:** Flux analysis to select the gene amplification targets for enhanced fumaric acid production.

	Enzyme	Functional category	Folds
ENO	enolase	Glycolysis/Gluconeogenesis	1.44
GAPD	glyceraldehyde-3-phosphate dehydrogenase	Glycolysis/Gluconeogenesis	1.04
PGK	phosphoglycerate kinase	Glycolysis/Gluconeogenesis	1.04
PGM	phosphoglycerate mutase	Glycolysis/Gluconeogenesis	1.44
PYK	pyruvate kinase	Glycolysis/Gluconeogenesis	1.49
TPI	triose-phosphate isomerase	Glycolysis/Gluconeogenesis	1.04
PC	Pyruvate carboxylase	Anaplerotic reactions	1.82
ACONTm	Aconitate hydratase	Citric Acid Cycle	0.99

### Effect of RoPYC Overexpression on FA Production

As an anaplerotic enzyme, cytosolic pyruvate carboxylase catalyzes the conversion of pyruvate to oxaloacetate. Kenealy et al. found high pyruvate carboxylase activity in a Rhizopus oryzae strain under aerobic conditions [3]. Thus, we investigated the effect of RoPYC overexpression, which encodes pyruvate carboxylase of R. oryzae, on FA production.

**Figure 4:**
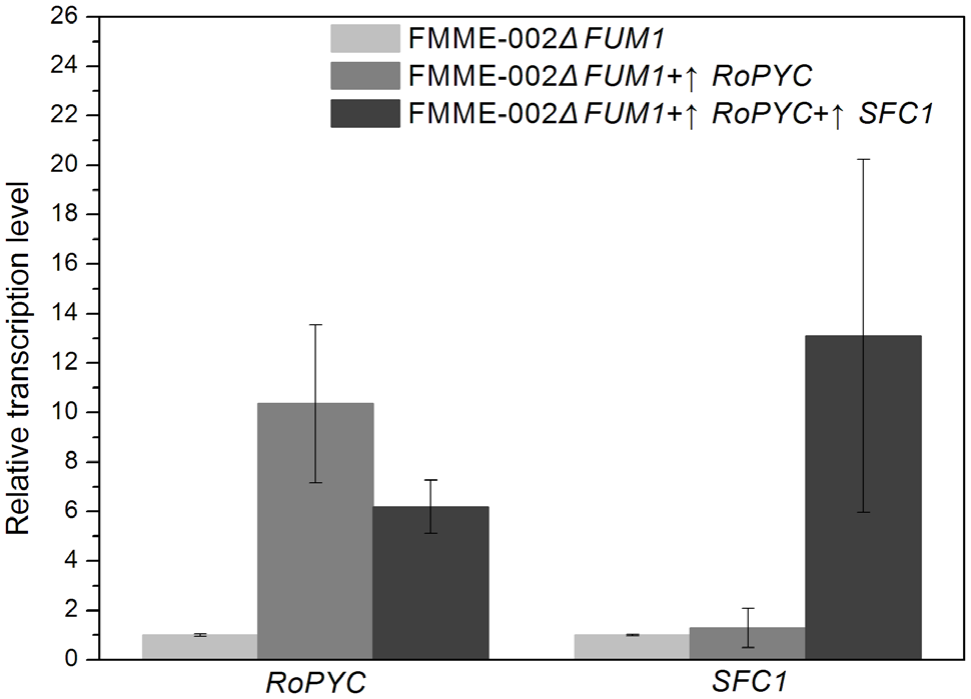
Relative gene expression levels. *RoPYC* and *SFC1* in the mutant strain FMME-002Δ*FUM1*, FMME-002Δ*FUM1*+↑*RoPYC* and FMME-002Δ*FUM1*+↑*RoPYC*+↑*SFC1*, Relative transcription levels were normalized to the transcription level of the β-*ACTIN* gene, which was taken as 1. The presented values are averages of three independent experiments; the error bars indicate standard deviations.

**Figure 5:**
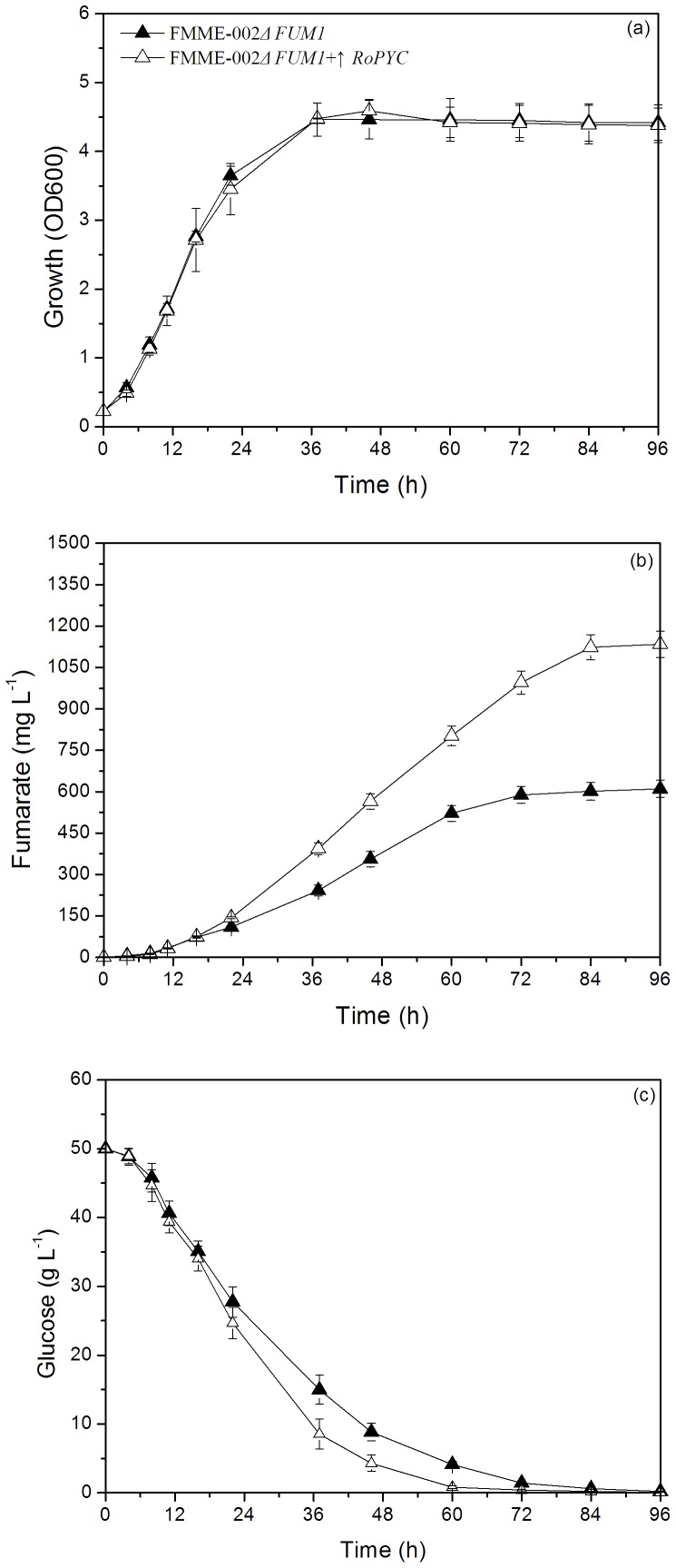
The effect of *RoPYC* over-expression on fermentation profile of engineered strain. Closed triangle represents the control strain FMME-002Δ*FUM1*, open triangle represents the mutant strain FMME-002Δ*FUM1*+↑*RoPYC*. (a): growth, (b): fumaric acid, (c) : glucose.

To test the hypothesis provided by the model, *RoPYC* overexpression in *S. cerevisiae* was investigated. The gene expression level of *RoPYC* was increased by 10-fold, as detected by quantitative real-time PCR (QT-PCR) (Figure 4). As shown in Figure 5, introduction of the *RoPYC* gene had a minimal effect on growth. However, the mutant FMME-002Δ*FUM1*+↑*RoPYC* accumulated FA up to a concentration of 1134±48 mg L^–1^ within 96 h, representing an increase in FA of 86% compared with the parent strain. The rate of glucose consumption improved and the FA yield also increased when the *RoPYC* gene was introduced.

### Improved FA Yield by Coexpression of RoPYC and SFC1

Efficient export of FA is also important to further enhance its production; on the contrary, insufficient transport capacity might impede efficient FA production. It has been reported that SFC1 encodes a succinate–fumarate transporter in S. cerevisiae [21]–[23].

**Figure 6:**
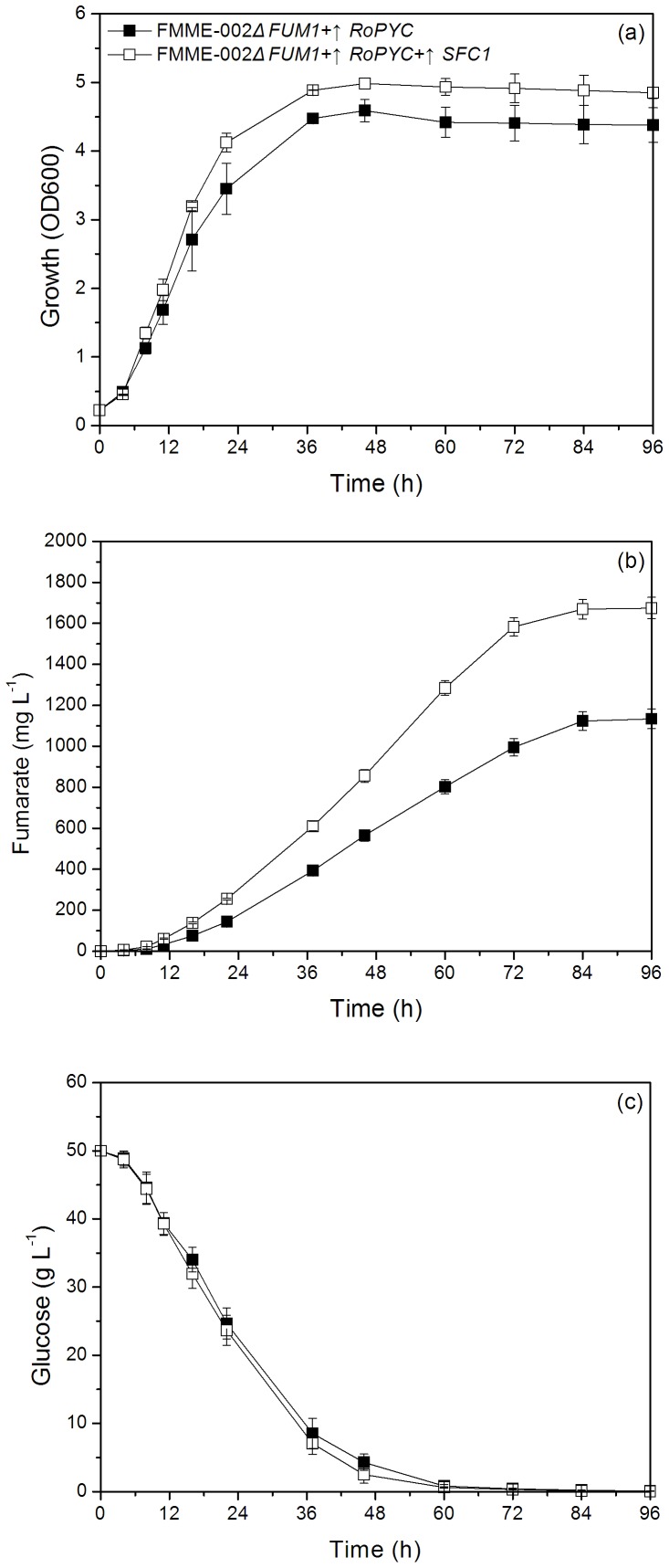
The effect of *SFC1* and *RoPYC* co-expression on fermentation profile of engineered strain. Closed square represents the control strain FMME-002Δ*FUM1*+↑*RoPYC*+ pY26TEF-GPD, open square represents the mutant strain FMME-002Δ*FUM1*+↑*RoPYC*+↑*SFC1*. (a): growth, (b): fumaric acid, (c): glucose.

Therefore, we investigated the potential of constitutive coexpression of RoPYC and SFC1 to further improve FA production. The gene expression levels of RoPYC and SFC1 were increased by 6-fold and 13-fold respectively (Figure 4). Results showed that additional introduction of SFC1 in the RoPYC overexpression strain lead to a slight increase in growth and glucose consumption and a significant improvement in FA production. The maximum FA concentration obtained was 1675±52 mg L^–1^ at 96 h, representing a 47.6% increase in comparison with the engineered strain FMME-002ΔFUM1+↑RoPYC. However, as shown in Figure 6, no apparent difference was observed in glucose consumption rate between these two strains.

Thus, metabolic engineering of S. cerevisiae based on GSMM analysis and transporter engineering successfully yielded a genetically defined FA overproducer.

## Discussion

None of the natural fumarate-producing microorganisms seem to be suitable for large-scale commercial production although high FA yields have been obtained [2], [3]. *S. cerevisiae* is an excellent platform for biologically based chemicals such as organic acids. The aim of the present study was to construct a genetically engineered *S. cerevisiae* strain that can produce FA. First, the target gene *FUM1* was identified in an extensive literature search and then FBA was used to predict the effect of *FUM1* disruption using the *S. cerevisiae i*ND750 GSMM. The simulated results revealed that *FUM1* deletion could lead to FA accumulation with only a slight influence on cell growth (∼1.95% lower). Then gene deletion was carried out and engineered *S. cerevisiae* cells produced FA at concentrations up to 610±31 mg L^–1^. Meanwhile, cell growth and glucose consumption were slightly lower compared to the parent strain, in accordance with the simulated result. Simulated results also showed that pyruvate carboxylase could be one of the factors limiting higher FA production, and an improved FA yield was obtained when the *RoPYC* gene was introduced. Furthermore, a significant improvement in FA production was achieved when the *SFC1* gene was introduced. The final FA concentration obtained was 1675±52 mg L^–1^. Thus, the engineered strain provides a potential new route for FA production. However, the concentration and yield are low in comparison with *R. oryzae* [2], so further work is required before this approach is economically feasible.

The number of GSMMs available is increasing sharply [24]. Because a GSMM represents nearly all the metabolic activities of an organism, it can be of great help in understanding metabolism on a global level [25]. Thus, GSMMs are widely used in metabolic engineering [26], [27] and can be used to predict and evaluate genetic manipulations in advance (dry experiments) when combined with certain algorithms [18], [28]. This can greatly improve the efficiency and directionality of metabolic engineering in various phases by predicting gene targets to be manipulated throughout the whole cellular network. In *S. cerevisiae*, metabolic engineering strategies aided by GSMM have led to improved production of various metabolites such as bioethanol, purine, proline/pyrimidines and vanillin [29]. In addition to direct improvements in production capacity, GSMMs can also be used to predict cellular properties or phenotypic traits such as growth and glucose consumption. In previous studies, growth behavior, and ethanol, succinate, citrate and fumarate concentrations were determined in various media (rich and minimal media) under aerobic and anaerobic conditions [11], [30], but the effect of *FUM1* deletion or fumarase deficiency on fermentation profiles (growth and glucose uptake rate) has not been studied in detail. In the present study, the phenotypic trait of slightly lower growth caused by *FUM1* deletion in *S. cerevisiae* was successfully predicted by FBA analysis; this trait is important for metabolic engineering because unwanted side effects can be induced. Metabolic models are also useful in identifying targets for further strain improvement. We identified pyruvate carboxylase as a factor restricting higher FA yield. A higher FA yield was obtained by increasing the flux through pyruvate carboxylase. The increased flux induced by overexpression of pyruvate carboxylase is linked to increased transport of cytosolic oxaloacetate into mitochondria and supply to oxidative reactions [31]. When pyruvate carboxylase and the transporter encoded by *SFC1* were coexpressed, a higher growth rate and FA yield were obtained, which suggests that insufficient FA export is another controlling step that would lead to higher FA production in steady-state metabolism.

The model showed some restrictions; however, the physiological characteristics observed for engineered organisms can be used to update the model. In the present study, there was very good accordance between *in silico* predictions and experimental results. The discrepancy between experimental and predictive yields was primarily caused by lack of model knowledge for yeast metabolism, regulatory mechanisms and feedback inhibition, which requires specific further experimental investigation.

In conclusion, the metabolic pathways in *S. cerevisiae* were rationally engineered for FA production with the aid of *in silico* simulations. The strategy described here can be useful for improved production of organic acids and other metabolites by direct microbial fermentation from renewable resources.

## Materials and Methods

### Strains and Plasmids

The strains and plasmids used are listed in Table 2. All yeast strains used were derived from strain *S. cerevisiae* CEN.PK2-1C (MATa ura3-52 leu2-3,112 trp1-289 his3Δ MAL2-8c SUC2), which was obtained from Euroscarf (Frankfurt, Germany). Plasmid pUG27 was constructed by replacing the *kanMX* marker in pUG6 with the his5^+^ marker from pFA6a-HIS3MX6 (which complements the *S. cerevisiae his3* mutation) using *Bgl*II and *Sac*I restriction sites, as previously described [32]. *Pyrobest* DNA polymerase for PCR was purchased from TaKaRa Biotechnology (Dalian, China).

**Table 2:** Strains and plasmids used in this study.

Strains and plasmids	Relevant characteristics	Source
Strains		
FMME-002	MATa; reference strain	Euroscarf
FMME-002Δ*FUM1*	MATa *fum1*::*lox*	This study
FMME-002Δ*FUM1*+↑*RoPYC*	MATa *fum1*::*lox*{ pY15TEF1-*RoPYC* }	This study
FMME-002Δ*FUM1*+↑*RoPYC*+↑*SFC1*	MATa *fum1*::*lox*{pY15TEF1-*RoPYC*,pY26TEF-GPD-*SFC1*}	This study
Plasmids		
pFA6a-HIS3MX6	Amp,*K.lactis* HIS3	Lab collection
pUG27	Amp,*K.lactis* HIS3,loxp	This study
pSH47	Amp,GAL1-cre, URA3	Prof. Hegemann JH
pY15TEF1	Amp,LEU2	Lab collection
pY26TEF-GPD	2 µm *URA3*, P_GPD/_T_CYC1_, P_TEF/_T_ADH1_	Lab collection
pY15TEF1-*RoPYC*	Amp,LEU2,P_TEF1_- *RoPYC*	This study
pY26TEF-GPD-*SFC1*	2 µm *URA3*, P_GPD/_T_CYC1_, P_TEF/_T_ADH1_, P_GPD_-*SFC1*	This study

The Cre-expressing plasmid pSH47 was used for marker rescue [33]; the vector carries the cre-recombinase gene to remove the *kanMX* gene flanked by *lox*P sites, then the Cre plasmid pSH47 is removed from this yeast strain. Primers used in confirming the loss of selectable marker are listed in Table 3.

**Table 3:** Primers used in this study.

Primers	Sequence (5′-3′)	Usage
45-F(*FUM1*)	GAAATTCCATAAAGTCTAACTATTAAACGGATAAGAGATACAATC-CAGCTGAAGCTTCGTACGC	Disruption of *FUM1*
45-R(*FUM1*)	TTATTTAGGACCTAGCATGTGTTCAGGAACAACCCATTCATCAAA-GCATAGGCCACTAGTGGATCTG	
A(*FUM1*)	AATCTTCATCACTGTTGTAGACGTT	Confirming the loss of the HIS marker
B(*FUM1*)	GTTTTCAAAGAATGTGCCACTC	
C(*FUM1*)	TGCAAGTCATGGGTAACAATGC	
D(*FUM1*)	CTTAATAGGACTCAAATCGTGATGG	
B-M	GCCTGCTTGAATGCAATACC	
C-M	CACGAAGGGAGTGTTGTAAAGAG	
F(*RoPYC*)	ATGCCTGCTGCACCAGTAC	Cloning of *RoPYC*
R(*RoPYC*)	TTAGGCTTCCTCTTTGACAACC	
SpeI-F(*RoPYC*)	GGACTAGT-ATGCCTGCTGCACCAGTAC	Construction of pY15TEF1-*RoPYC*
SalI-R(*RoPYC*)	ACGCGTCGAC-TTAGGCTTCCTCTTTGACAACC	
F(*SFC1*)	ATGTCTCAAAAAAAGAAGGCTTC	Cloning of *SFC1*
R(*SFC1*)	CTACTTTAATGGCTTTGGCTTTG	
BamHI-F(*SFC1*)	CGGGATCC-ATGTCTCAAAAAAAGAAGGCTTC	Construction of pY26TEF-GPD-*SFC1*
HindIII-R(*SFC1*)	CCCAAGCTT-CTACTTTAATGGCTTTGGCTTTG	

### Literature Mining Method

To identify genes associated with FA production in *S. cerevisiae*, a literature search was carried out. The literature database consisted of articles downloaded from public online databases, including ScienceDirect (http://www.sciencedirect.com/), Springer Link(http://www.springerlink.com/), PubMed(http://www.ncbi.nlm.nih.gov/pubmed/), and ISI Web of Science (http://www.isiknowledge.com). Manual surveys of these papers were then carried out to obtain objective information.

### Simulation of Single Gene Deletion

The impact of gene deletion on cell growth and FA production was evaluated *in silico* by FBA using the *S. cerevisiae i*ND750 GSMM. To simulate single gene deletion, the flux of the reactions in *i*ND750 catalyzed by target genes was set to zero (i.e., no flux). Then FBA was carried out to predict optimum growth in minimum medium in which D-glucose was the sole carbon source. The rate of D-glucose uptake was set to 10 mmol g^–1^ DCW h^–1^ and the biomass equation was taken as the objective function. The rates of growth and FA production were simulated and compared with values for the non-modified *i*ND750 model under the same culture conditions. A robustness analysis of the rates of D-glucose uptake and growth between the original and modified models was also conducted. FBA simulations were performed using COBRA Toolbox v2.0 in MATLAB 2010b, with GLPK as the linear programming solver [34].

### Construction of *S. cerevisiae* Deletion Strains

Deletion of the *FUM1* gene (1467 bp) was performed via a one-step inactivation method [33]. The vector pUG27 was used to delete this gene in *S. cerevisiae* CEN.PK2-1C. After plasmid preparation, a fragment of pUG27 was amplified by PCR to obtain a cassette consisting of *loxP*-*his5^+^*-*loxP*. Primers were constructed (Table 3) to fuse 5′ and 3′ coding sequences of *FUM1* and sequences of the *loxP* regions of the pUG27 vector. The resulting PCR product comprised the *his5^+^* gene, *loxP* sites and *FUM1* homologous regions for integrative transformation in *S. cerevisiae* CEN.PK2-1C. Homologous recombination in yeast led to deletion of the target gene. SC selection medium was prepared by adding a mixture of amino acids, purines and pyrimidines to Difco yeast nitrogen base, and histidine was omitted to select for positive clones. Then the His marker was removed via transformation with pSH47. The vector carried the cre-recombinase gene to remove the *his5^+^* gene flanked by *loxp* sites induced by 2% D-galactose. The Cre plasmid was subsequently removed from the yeast strain.

### Plasmid Construction and Transformation

cDNA of *R. oryzae* NRRL1526 (ATCC 10260) was obtained as previously described [10]. Then PCR primer pairs covering the entire open reading frame of the *RoPYC* gene were designed according to GenBank sequences for *R. oryzae* using Primer Premier v5.0 software (Table 3). Thermal cycling was carried out as follows: the initial denaturation step was at 94°C for 5 min, followed by 29 cycles of denaturation at 94°C for 30 s, annealing at 52°C for 30 s, and extension at 72°C for 3.6 min, with a final single extension step at 72°C for 10 min. Then a 3540-bp fragment of the *RoPYC* gene was amplified by nested PCR. The sequence of this gene fragment was submitted to GenBank under accession number HM130700.1. Gene-specific primers were designed to amplify *RoPYC*, and *RoPYC* was then amplified by PCR using cDNA of *R. oryzae* NRRL1526 as a template. The resultant PCR fragment of *RoPYC* and expression vector pY15TEF1 were digested with SpeI and SalI and ligated together to create the pY15TEF1-*RoPYC* plasmid. The *S. cerevisiae SFC1* gene was amplified by PCR from chromosomal DNA of CEN.PK2-1C using the primers BamHI-F(*SFC1*) and HindIII-R(*SFC1*). The PCR fragment and pY26TEF-GPD vector were digested with BamHI and HindIII and ligated to create pY26TEF-GPD-*SFC1*.

Plasmids were introduced into yeast cells using a Frozen-EZ yeast transformation II kit (Zymo Research, Orange, CA, USA) according to manufacturer’s protocol. Transformants were selected on agar plates of synthetic complete (SC) selection medium lacking leucine and uracil as auxotrophic markers.

### Transcriptional Analysis

For RNA extraction, flask culture cells in early stationary phase were harvested by centrifugation (8000 rpm at 4°C for 5 min). Total RNA was isolated using an RNAprep pure plant kit according to the manufacturer’s instructions. The amount of isolated RNA was determined photometrically (optical density at 260 nm [OD_260_] of 1 equals 40 µg mL^–1^ RNA). Reverse transcription was carried out using an iScript™ cDNA Synthesis Kit (Bio-Rad) according to the manufacturer’s instructions. Amplification of cDNA via quantitative real-time PCR was carried out using an iTaq™ Universal SYBR Green Supermix Kit (Bio-Rad). The reaction mix consisted of 10 µL iTaq™ Universal SYBR Green Supermix (2×), 1 µL each of forward and reverse primers (10 µmol), 2 µL of cDNA (50 ng), and 6 µL H_2_O. Primers used in the transcriptional analysis are listed in Table 4. Amplication and detection of specific products were performed on a BIO-RAD® CFX96 system (Bio-Rad) according to following program: 95°C for 30 s, and 39 cycles of 95°C for 5 s, 55°C for 12 s. Data analysis was performed using the second derivative method, and expression levels were normalized to expression of the *ACT1* reference gene. Each sample was tested in triplicate in a 96-well plate (Bio-Rad, Hercules, CA, USA).

**Table 4:** Primers used in the transcriptional analysis.

Primer	Sequence, 5′-3′	Analyzed gene
F(*RoPYC*)	TGTTGAAGCCACCATCTG	*RoPYC*
R(*RoPYC*)	GCACGGATACTGAAGACC	
F(*SFC1*)	GGCAAGGCAAGCAACTAATCAG	*SFC1*
R(*SFC1*)	CCAATAGCACCCGAAATCAAACC	
F(*ACT*)	TTATTGATAACGGTTCTGGTATG	β*-ACTIN*
R(*ACT*)	CCTTGGTGTCTTGGTCTAC	

### Medium and Batch Fermentations

Luria-Bertani (LB) medium (5 g L^–1^ yeast extract, 10 g L^–1^ tryptone and 10 g L^–1^ NaCl) was used for plasmid purification from *E. coli* JM109. The fermentation medium (pH 5.0) contained (per liter) 50 g of glucose, 2 g of CO(NH_2_)_2_, 5 g of KH_2_PO_4_, 0.8 g of MgSO_4_⋅7H_2_O, and 10 mL of trace metal solution (0.2 g L^–1^ MnCl_2_⋅4H_2_O, 2 g L^–1^ FeSO_4_⋅7H_2_O, 2 g L^–1^ CaCl_2_⋅2H_2_O, 0.05 g L^–1^ CuSO_4_⋅5H_2_O, 0.5 g L^–1^ ZnCl_2_). In flasks, 40 g L^–1^ CaCO_3_ (dry-heat sterilized at 160°C for 30 min) was used as a buffer for the medium. Flasks were incubated at 30°C in an orbital shaker at 200 rpm. YPD medium (10 g L^–1^ yeast extract, 20 g L^–1^ peptone, 20 g L^–1^ glucose) was used for seed culture. The seed culture was inoculated with yeast growing well on an agar slant and was incubated for 24 h in a 250-mL flask containing 20 mL of seed medium. The broth was centrifuged, the pellet was resuspended in isometric fresh fermentation medium, and the cell suspension was inoculated into a 250-mL shaker flask containing 50 mL of fermentation medium.

### Analytical Methods

Cell growth was monitored by measuring the absorbance at 600 nm (OD_600_) spectrophotometrically. Cell concentration, defined as grams of dry cell weight (g DCW) per liter, was calculated from a standard curve relating OD_600_ to dry weight (1 OD_600_ = 0.35 g DCW L^–1^). Extracellular concentrations of fumaric acid, ethanol and glucose were determined by HPLC using an Aminex HPX-87H column (Bio-Rad) eluted with 0.0275% (v/v) H_2_SO_4_ at a flow rate of 0.6 mL min^–1^ at 35°C. Fumaric acid was monitored using an Agilent (Santa Clara, CA, USA) 1200 series DAD detector at 210 nm. Ethanol and glucose were monitored using a 1200 series Agilent refractive index detector.

### Confirmation of FA Biosynthesis by FT-IR and NMR

Cell cultures of the engineered strain were centrifuged and the supernatant was adjusted to pH 1.0 by addition of HCl. Following acidification, fumaric acid precipitated out of the solution and was recovered by drying in a rotary dryer. The sample obtained was processed in parallel with a fumaric acid reference standard for FT-IR and ^1^H and ^13^C NMR analyses. FT-IR spectra were recorded on a Nicolet Nexus 470 spectrophotometer with a DTGS detector. Fumarate samples and the reference standard were diluted in KBr and measured in transmittance mode over the spectral range 400–4000 cm^−1^. ^1^H NMR (400 MHz, D_2_O, 25°C) and ^13^C NMR (100 MHz, D_2_O, 25°C) spectra were recorded on an Avance III 400-MHz digital NMR spectrometer.

## Supporting Information

Figure S1 **PCR analysis to confirm correct integration of the HIS marker gene disruption cassette at the** ***YPL262W*** **locus and confirm the loss of selectable marker.** The size of the expected PCR products is given below each lane. Wild type, nontransformed wild type yeast strain; Mutant I, mutant yeast strain carrying the HIS marker gene disruption cassette; Mutant II, mutant yeast strain without marker gene.
(TIF)

Figure S2 **The IR spectra of fumaric acid.** (a) sample; (b) the fumaric acid standard.
(TIF)

Figure S3 **The ^1^H and ^13^C NMR spectrum of sample and the fumarate standard.** (a) ^1^H spectrum of the fumarate standard; (b) ^13^C spectrum of the fumarate standard; (c) ^1^H spectrum of sample; (d) ^13^C spectrum of sample.
(RAR)

Table S1 **The central metabolic flux of CEN.PK2-1C** ***ΔFUM1*** **was analyzed using** ***i*** **ND750 and an FBA algorithm.** The genome-scale analysis was divided into three types of intracellular flux profiles occur: increased, decreased and irregular. The “increased” represents that the flux increases when FA production successively increased, the “decreased” represents that the flux decreases when FA production successively increased, and the “irregular” represents that the flux is unchanged or ruleless when FA production successively increased.
(XLSX)
