# Supplementary figures and images for: Fumaric Acid Production in Saccharomyces cerevisiae by In Silico Aided Metabolic Engineering
Source: PLoS One. 2012 Dec 26;7(12):e52086. doi: 10.1371/journal.pone.0052086 (PMC3530589; doi:10.1371/journal.pone.0052086)

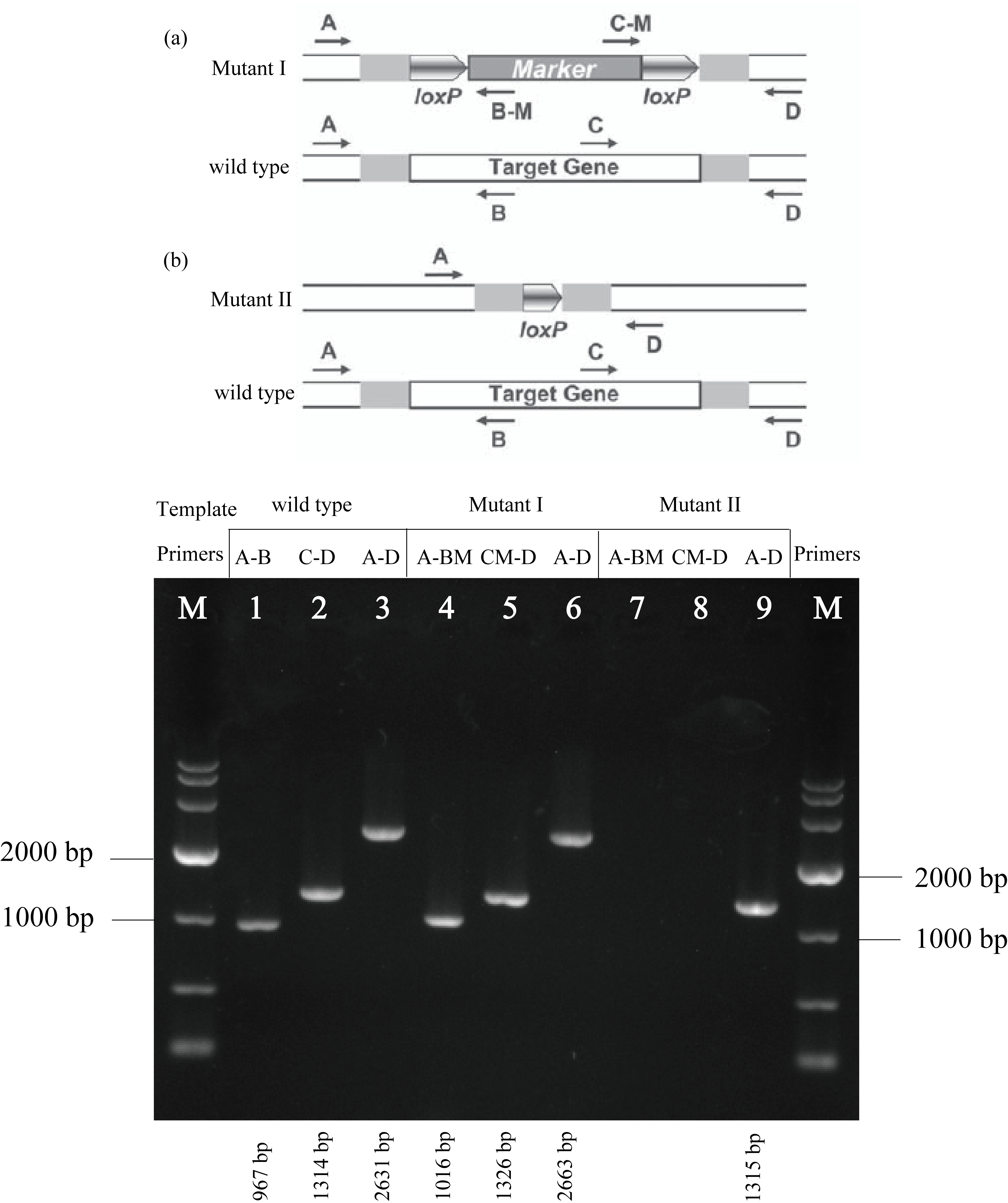

Supplement: Figure S1 — PCR analysis to confirm correct integration of the HIS marker gene disruption cassette at the YPL262W locus and confirm the loss of selectable marker. The size of the expected PCR products is given below each lane. Wild type, nontransformed wild type yeast strain; Mutant I, mutant yeast strain carrying the HIS marker gene disruption cassette; Mutant II, mutant yeast strain without marker gene. (TIF) [file pone.0052086.s001.tif]

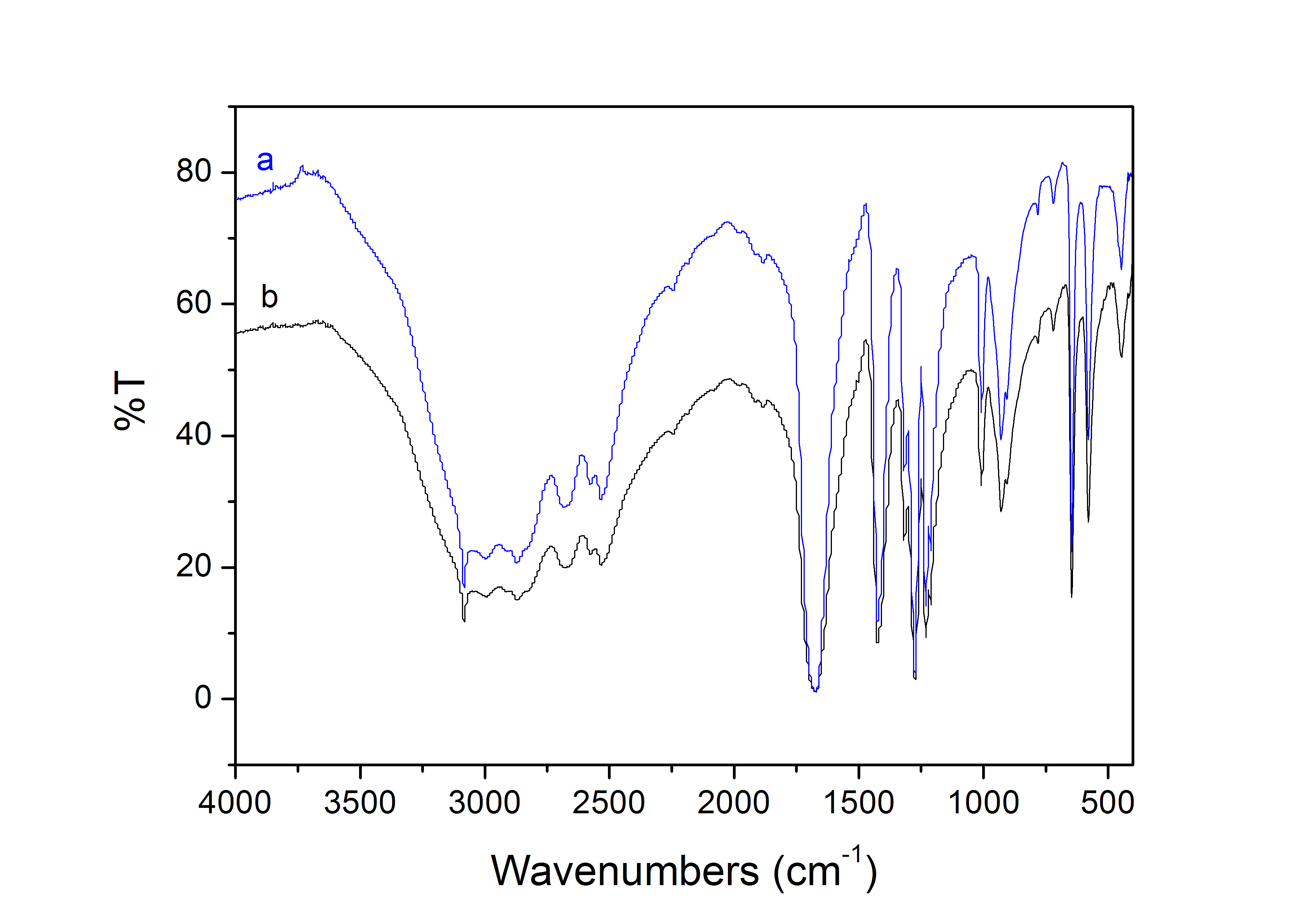

Supplement: Figure S2 — The IR spectra of fumaric acid. (a) sample; (b) the fumaric acid standard. (TIF) [file pone.0052086.s002.tif]
